# 

**Published:** 1907-12-21

**Authors:** 


### CONTENTS.

| | PAGE |
|---|---|
| **King Edward's Hospital Fund** | 303 |
| **Further Notes on Compulsory Notification** | 304 |
| **Annotations—** | |
| Medical Students and their Critics—Pneumococcus Infections—Society for the Study of Disease in Children | 305 |
| **Medical Opinion and Movement** | 306 |
| **Hospital Clinics—** | |
| The Ultimate Results of Inoperable Gynæcological Cases. By A. E. Giles, B.Sc., M.D., F.R.C.S. | 307 |
| **Clinical Points—** | |
| The Smegma Bacillus and Urinary Tuberculosis | 309 |
| **Points in Diagnosis—** | |
| Justus' Test for Syphilis | 310 |
| **Points in Surgery—** | |
| Dislocation of the Shoulder | 311 |
| Some Diseases of the Male Genital System | 312 |
| **Laryngology and Rhinology—** | |
| Some Effects of Adenoids | 313 |
| **Points in Pathology—** | |
| Endothelial Tumours | 314 |
| **Public Health and Hygiene—** | |
| The "Rideal-Walker" Method of Standardising Disinfectants | 315 |
| The "Ad Hoc" Principle in Local Government | 316 |
| **Therapeutics—** | |
| Prescriptions and Dispensing | 317 |
| **Practical Notes on Diagnosis and Treatment** | 318 |
| **"The Hospital" Medical Book Supplement, No. 2** | 319 |
| **Hospital Administration—** | |
| King Edward's Hospital Fund for London | 323 |
| The Hospital Sunday Fund | 327 |

